# Relationships between Indicators of Lower Extremity Artery Disease and miRNA Expression in Peripheral Blood Mononuclear Cells

**DOI:** 10.3390/jcm11061619

**Published:** 2022-03-15

**Authors:** Daniel P. Zalewski, Karol P. Ruszel, Andrzej Stępniewski, Dariusz Gałkowski, Marcin Feldo, Janusz Kocki, Anna Bogucka-Kocka

**Affiliations:** 1 Chair and Department of Biology and Genetics, Medical University of Lublin, 4a Chodźki St., 20-093 Lublin, Poland; anna.kocka@umlub.pl; 2 Department of Clinical Genetics, Chair of Medical Genetics, Medical University of Lublin, 11 Radziwiłłowska St., 20-080 Lublin, Poland; karol.ruszel@umlub.pl (K.P.R.); janusz.kocki@umlub.pl (J.K.); 3 Ecotech Complex Analytical and Programme Centre for Advanced Environmentally Friendly Technologies, University of Marie Curie-Skłodowska, 39 Głęboka St., 20-612 Lublin, Poland; andrzej.stepniewski@umcs.pl; 4 Department of Pathology and Laboratory Medicine, Rutgers-Robert Wood Johnson Medical School, One Robert Wood Johnson Place, New Brunswick, NJ 08903-0019, USA; galkowd@fastmail.fm; 5 Chair and Department of Vascular Surgery and Angiology, Medical University of Lublin, 11 Staszica St., 20-081 Lublin, Poland; martinf@interia.pl

**Keywords:** miRNA, miRNA expression, lower extremity artery disease, peripheral arterial disease, atherosclerosis, ankle brachial index, claudication, next generation sequencing

## Abstract

Lower extremity artery disease (LEAD) is an underdiagnosed and globally underestimated vascular disease caused by the progressive and chronic formation of atherosclerotic plaques in the arteries of the lower limbs. Much evidence indicates that the abnormal course of pathophysiological processes underlying LEAD development is associated with altered miRNA modulatory function. In the presented study, relationships between miRNA expression and clinical indicators of this disease (ABI, claudication distance, length of arterial occlusion, Rutherford category, and plaque localization) were identified. MiRNA expression profiles were obtained using next-generation sequencing in peripheral blood mononuclear cells (PBMCs) of 40 LEAD patients. Correlation analysis performed using the Spearman rank correlation test revealed miRNAs related to ABI, claudication distance, and length of arterial occlusion. In the DESeq2 analysis, five miRNAs were found to be dysregulated in patients with Rutherford category 3 compared to patients with Rutherford category 2. No miRNAs were found to be differentially expressed between patients with different plaque localizations. Functional analysis performed using the miRNet 2.0 website tool determined associations of selected miRNAs with processes underlying vascular pathology, such as vascular smooth muscle cell differentiation, endothelial cell apoptosis, response to hypoxia, inflammation, lipid metabolism, and circadian rhythm. The most enriched functional terms for genes targeted by associated miRNAs were linked to regulation of the cell cycle, regulation of the transcription process, and nuclear cellular compartment. In conclusion, dysregulations of miRNA expression in PBMCs of patients with LEAD are indicative of the disease and could potentially be used in the prediction of LEAD progression.

## 1. Introduction

Lower extremity artery disease (LEAD) belongs to the broad group of peripheral arterial diseases (PAD) manifested by the formation of atherosclerotic plaques in peripherally located arteries (e.g., carotid, mesenteric, extremity, or renal arteries). In LEAD, atherosclerotic lesions develop in the arteries of the lower limbs, impairing blood supply and causing ischemia-related symptoms, such as intermittent claudication, ischemic rest pain, and leg ulceration [1].

More than 200 million people worldwide and about 40 million people in Europe are affected by LEAD. The overall global prevalence of this disease exceeds 10% in people older than 70 years [2,3]. Age, smoking, diabetes, hypertension, and hypercholesterolemia have been identified as the major risk factors for LEAD [1,3,4,5]. The most severe consequence of LEAD is critical limb ischemia threatening amputation, which is caused by complete obstruction of the artery and affects approximately 11% of patients with LEAD [1,6,7]. Due to the fact that this disease is considered an underdiagnosed and undertreated condition, early and proper detection of affected individuals is crucial to control the disease and reduce its negative impact on the global community [8].

The first-line indicator used to diagnose LEAD is the ankle brachial index (ABI), which is the ratio of systolic blood pressure in the ankle area (on the dorsal artery of the foot or tibial arteries) to the systolic pressure measured on the brachial artery. The normal ABI value is in the range of 1.0–1.4, while a value below 0.9 indicates a disturbance of blood flow in the lower limbs. In turn, ABI higher than 1.4 suggests arterial stiffening, which is primarily caused by calcification [1,5,8,9]. Both abnormally low and high ABI is an indicator of a higher risk of cardiovascular events and mortality [1,9,10,11]. ABI measurement is cost-effective, relatively easy to conduct and capable of detecting asymptomatic cases; therefore, it is recommended for use in screening studies aimed at describing the global distribution of LEAD [5].

Another useful indicator to describe the advancement of LEAD is claudication distance, which is the maximum distance a patient can walk until increased pain prevents further walking. This indicator enables the evaluation of walking impairment in patients with LEAD and is typically obtained during treadmill tests [1]. Lower values of the claudication distance indicate more advanced arterial occlusion. The measurement of the claudication distance has special utility in the cases of borderline ABI (0.9–1.0) and normal ABI with clinical manifestations suggesting LEAD [12].

However, despite the high usability of ABI and claudication distance examinations, the most conclusive tool in LEAD diagnosis is imaging techniques, including vascular duplex ultrasound and angiography performed with computed tomography or magnetic resonance [1,12,13].

To assess the progress of LEAD, the results of diagnostic tests and the clinical presentations observed in this disease are categorized according to the Fontaine or Rutherford classifications. Fontaine classification includes stages I–IV and Rutherford classification includes grades 0–III (divided into seven categories) according to the severity of the disease [1,12].

The progression of LEAD is caused by the continuous chronic growth of atherosclerotic plaques in the lower limb arteries, promoted by the abnormal course of physiological processes such as inflammation, angiogenesis, endothelial function, and vascular smooth muscle cell (VSMC) proliferation and apoptosis [14,15,16,17]. Dysregulations of these processes result from imbalance of variety of molecular factors, including microRNA (miRNA), which is a group of small, non-coding RNAs involved in regulation of gene expression at the post-transcriptional level. MiRNA molecules incorporated into protein complexes can bind to mRNA strands, primarily causing repression or degradation of mRNA translation [18,19,20,21]. Many studies have evidenced an important role of miRNAs in the development of atherosclerosis [22,23,24,25] and PAD [26,27,28,29]. Differentially expressed miRNAs were reported in various tissues of patients with LEAD and were proposed as potential candidates for biomarkers of this disease [30,31,32,33,34,35,36]. Several circulating miRNAs have been shown to have potential for use as a diagnostic, prognostic, and therapeutic targets for peripheral atherosclerosis [28,37,38,39,40].

There is limited information about the relation between miRNA expression and clinical parameters of LEAD advancement, such as ABI, claudication distance, or Rutherford and Fontaine categories. In previous research, an increase in the expression level of miR-124-3p induced by ischemic conditions was negatively correlated with ABI [38]. Higher serum levels of miR-93 have previously been found to be positively associated with PAD severity measured by ABI scores [41]. Downregulation of miR-126 in plasma was associated with lower ABI and the occurrence of symptomatic peripheral artery disease in diabetic patients [42]. Reduced expression levels of miR-21, miR-92a, miR-126, and miR-143 were found in the circulatory exosomes of patients diagnosed with a median Rutherford category 2.5 compared to those of patients with a median Rutherford category 4 [43]. These findings suggest that dysregulations of miRNA expression could be an indicator of LEAD progression.

Therefore, in the presented study, we explore the relationships between alterations in whole miRNA-ome expression profiles and clinical indicators of LEAD progression such as ABI, claudication distance, length of arterial occlusion, Rutherford category, and arterial localization of atherosclerotic plaques. This study is based on miRNA expression datasets analyzed in our previous research regarding the identification of dysregulations in the miRNA regulatory network in patients with LEAD compared to healthy controls [34].

## 2. Materials and Methods

### 2.1. Study Participants

The study was performed in accordance with the Declaration of Helsinki and the study project was approved by the Bioethics Committee at the Medical University of Lublin (decision No. KE-0254/341/2015). The study group consisted of 40 patients with LEAD who participated in our previous study regarding identification of dysregulations in miRNA regulatory network in LEAD [34]. Characteristics of patients and inclusion procedure were provided in our previous paper [34]. Written informed consent was obtained from all study subjects.

### 2.2. miRNA Expression Datasets

MiRNA expression datasets were generated by sequencing small RNA fractions isolated from PBMCs samples collected from 40 patients with LEAD. Sequencing experiments were performed using Ion S5 XL System (Thermo Fisher Scientific, Waltham, MA, USA). The procedure of PBMC and RNA isolation, quality and quantity assessment of RNA, library preparation, sequencing, and primary analysis of raw sequencing data were previously described [34].

Data used in the current study contain raw reads counts for 2792 miRNA transcripts (collected in miRBase v21, http://www.mirbase.org) obtained for each LEAD subject. This expression data was deposited in the Figshare repository (https://doi.org/10.6084/m9.figshare.19164659.v2, accessed on 11 February 2022) along with data regarding healthy controls. Prior to analysis, expression data were filtered to remove miRNA transcripts with mean of raw reads counts ≤1 and was normalized using the DESeq2 method implemented in the DESeq2 1.34.0 package for R [44] (https://bioconductor.org/packages/release/bioc/html/DESeq2.html, accessed on 2 November 2021).

### 2.3. Data Analysis

Data analysis was performed using appropriate packages in R environment (version 3.6.3, https://www.r-project.org) using code described in corresponding reference manuals. The relationships between miRNA expression and continuous variables were investigated using correlation and simple linear regression. Correlation analysis was performed using the Spearman rank correlation test implemented in Hmisc package 4.5-0. (https://cran.r-project.org/web/packages/Hmisc/index.html, accessed on 5 September 2021). Multivariate linear regression models were constructed using lm() base function in R. The relationships between miRNA expression and categorical variables were analyzed using DESeq2 and ROC. DESeq2 analysis was carried out using the DESeq2 1.26.0 package and ROC analysis was performed using the pROC package 1.18.0 [45] (https://cran.r-project.org/web/packages/pROC/index.html, accessed on 15 December 2021).

The functional analysis of selected miRNAs was performed using the miRNet 2.0 online platform [46] (https://www.mirnet.ca, accessed on 22 December 2021) with a hypergeometric test applied to functional terms of “miRNA-Function“ category. Obtained relationships were visualized using Cytoscape v3.7.0 software (https://cytoscape.org/) [47]. The MiRNet 2.0 database was also used to identify targets for selected miRNAs and to perform functional analysis of identified targets with application of hypergeometric tests to Gene Ontology Biological Processing (GOBP), Gene Ontology Cellular Compartment (GOCC), Gene Ontology Molecular Function (GOMF), KEGG (Kyoto Encyclopedia of Genes and Genomes) and Reactome categories.

## 3. Results

### 3.1. The Study Group and Expression Dataset

The study group included 40 patients with LEAD. This group was previously studied to identify dysregulations of the miRNA regulatory network in LEAD by comparison with healthy controls [34]. The current study is a continuation of previous research and includes investigations of the relationships between miRNA expression and clinical characteristics of LEAD advancement. Investigated characteristics include ABI, claudication distance, length of arterial occlusion, Rutherford category, and plaque localization, measured during the qualification of patients for the previous study. Clinical and demographic data concerning the study group were provided in the previous study [34] and in an extended form are presented in Table 1.

The expression data used in the present study constitute miRNA expression profiles containing information about 2792 miRNA transcripts in PBMCs (peripheral blood mononuclear cells) of LEAD patients and generated using next-generation sequencing [34]. Prior to analysis, expression data were filtered to remove miRNA transcripts with mean of raw read counts ≤1, retaining information about the expression of 1958 miRNA transcripts. Furthermore, raw numeric values of read counts were normalized using the DESeq2 method. Normalized expression data were used to identify relationships between miRNA expression and patient characteristics associated with LEAD advancement.

### 3.2. Relationships between miRNA Expression and Continuous Characteristics of LEAD

**Figure 1:**
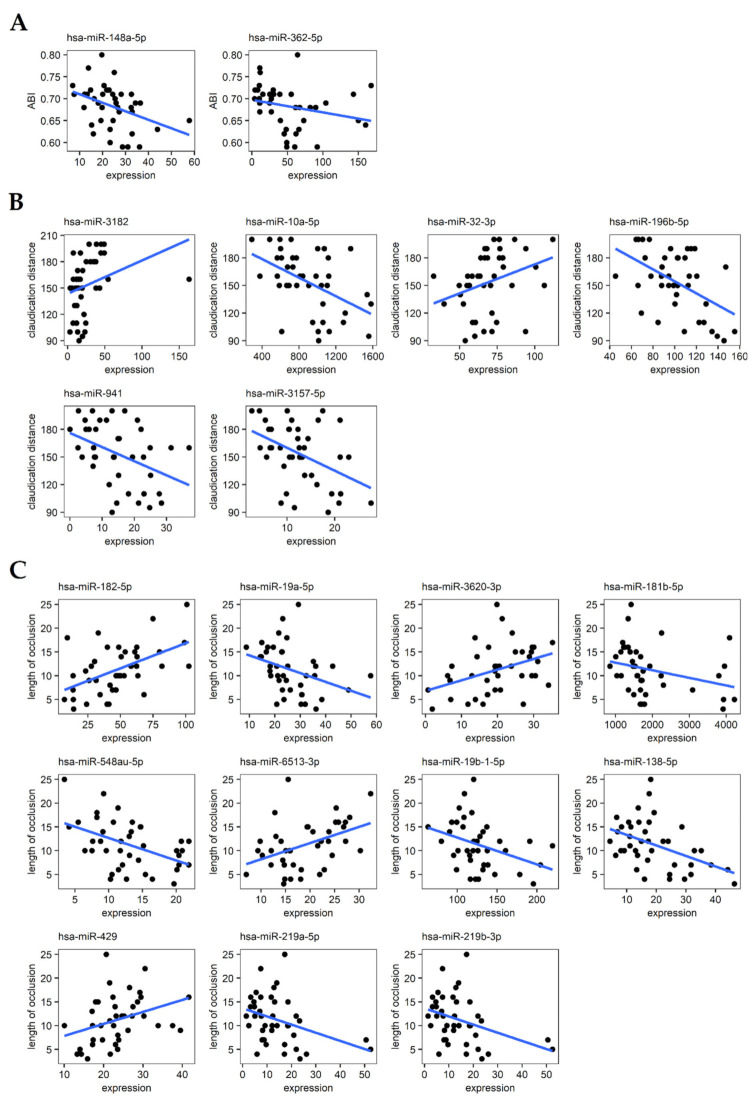
Scatter plots depicting correlations between 19 selected miRNAs and clinical parameters of LEAD progression including (**A**) ankle brachial index (ABI), (**B**) claudication distance, and (**C**) length of arterial occlusion. Blue lines indicate the trend line (fitting line of the simple linear regression model).

Correlation analysis was performed to find relationships between the expression of 1958 miRNAs and continuous-type parameters of LEAD progression (ABI, claudication distance, and length of arterial occlusion). The analysis was carried out using the Spearman rank correlation test with application of the absolute value of the Spearman correlation coefficient ≥0.4 and *p* value < 0.05 as a selection criteria. As a result, 9, 18, and 21 miRNA transcripts were found to be correlated with ABI, claudication distance, and length of occlusion, respectively (Table S1, Figures S1–S3). However, some of the correlated miRNA transcripts have very low expression (Figures S1–S3), and thus correlations with these miRNAs were evaluated to be not scientifically significant. Therefore, only the correlations regarding 19 miRNA transcripts with the average number of normalized counts >10 were selected as relevant (Table 2, Figure 1) and subjected to further analyses. The entire correlation results are provided in Supplementary File 2.

Multivariate linear regression analysis was used to further explore the relationships between miRNA expression and studied continuous parameters of LEAD progression. ABI, claudication distance, and length of arterial occlusion were set as response variables, and the expression of 19 relevantly correlated miRNAs were used as explanatory variables. To adjust the potential influence of demographical traits such as age, sex, body mass index (BMI), and smoking status, these variables were also included in the regression models. The results of the regression analysis, including the intercept values and the regression coefficients, are provided in Table 2.

### 3.3. Relationships between miRNAs Expression and Categorical Characteristics of LEAD

**Figure 2:**
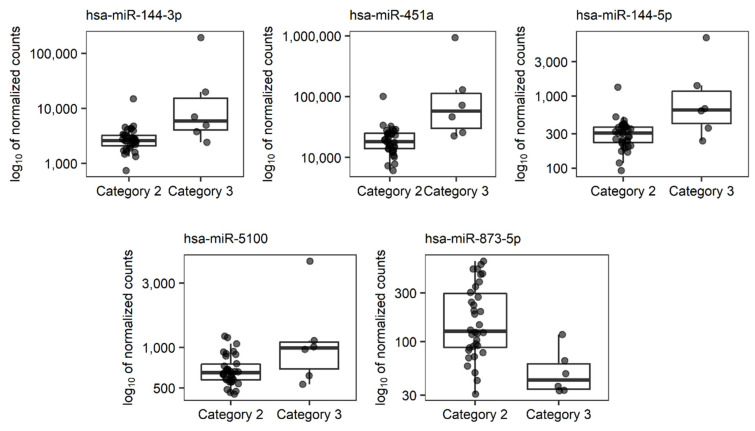
Boxplots presenting normalized expression of 5 selected miRNAs differentially expressed in patients diagnosed with Rutherford category 3 (Category 3) versus patients diagnosed with Rutherford category 2 (Category 2). In the boxplots, whiskers reach extreme samples inside the 1.5 inter-quartile range, boxes range between 25% and 75% quartile, and horizontal lines inside boxes mark median values.

Associations between the expression of 1958 studied miRNA transcripts and the categorical characteristics of LEAD (Rutherford category and localization of atherosclerotic plaques) were evaluated using differential expression analysis performed by DESeq2 method. Five miRNAs (hsa-miR-144-3p, -144-5p, -451a, -873-5p, and -5100) were found to be significantly differentially expressed (Benjamini-Hochberg FDR < 0.05) between 6 patients diagnosed with Rutherford category 3 and 34 patients diagnosed with Rutherford category 2 (Table 3, Figure 2, Supplementary File 2). The control plots generated for this analysis, including the MA plot and the histogram of *p* values, are presented in Figure S4. Identified associations were further analyzed using the ROC (Receiver Operating Characteristics) method, which showed good performance in the classification of the Rutherford category in the studied group of patients (Table 3, Table S2, Figure S5).

Regarding analysis of plaques arterial localization, a pairwise differential analysis of miRNA expression was performed using the DESeq2 method among 7 patients with iliac plaques (IL), 25 patients with femoral plaques (FEM), and 5 patients with popliteal plaques (POP). Three patients with plaques in more than one artery (refer to Table 1) were excluded from the analysis. The analysis included the following comparisons: IL vs. FEM, IL vs. POP, and POP vs. FEM. No miRNA transcript was differentially expressed with Benjamini-Hochberg FDR < 0.05 from any of the performed comparisons (Supplementary File 2). This result suggests an inability to differentiate plaque localization in lower limbs by miRNA expression of PBMCs specimens; however, further studies with larger groups of patients are required to validate this conclusion.

### 3.4. The Relationships between Selected miRNAs Associated with LEAD Indicators and Risk Factors, Cardiovascular Comorbidities, Results of Laboratory Tests, and Medication

The presented associations between expression of miRNAs and analyzed indicators of LEAD could be potentially affected by other characteristics related to the studied subjects, including risk factors, cardiovascular comorbidities, results of laboratory tests and medications (refer to Table 1). Therefore, the relationships between these characteristics and the expression of 2, 6, 11 and 5 miRNAs selected as related to ABI, claudication distance, length of arterial occlusion (Table 2) and Rutherford category (Table 3), respectively, were analyzed.

The Spearman rank correlation test was used to identify the relationships between these miRNAs and continuous variables, including age, BMI, blood morphology parameters (hemoglobin content, hematocrit levels, and counts of red blood cells, leucocytes, neutrophils, lymphocytes, monocytes, eosinophils, and basophils) and serum of creatinine and urea concentrations. The absolute value of the Spearman correlation coefficient ≥0.4 and *p* value < 0.05 were applied as a cut off criteria. The correlations that met these criteria are presented in Table 4 and Figure S6.

The DESeq2 method was used to identify the relationships between selected miRNAs and categorical variables, including sex, smoking status (former/current), risk factors (diabetes type 2 and hypertension), cardiovascular comorbidities (coronary artery disease, myocardial infarction, stroke/transient ischemic attack) and medication (see Table 1). As a result, 7 miRNAs were selected as significantly associated (Benjamini-Hochberg FDR < 0.05) with analyzed categorical characteristics (Table 5, Figure S7).

### 3.5. Functional Analysis of miRNAs Associated with LEAD Progression

**Figure 3:**
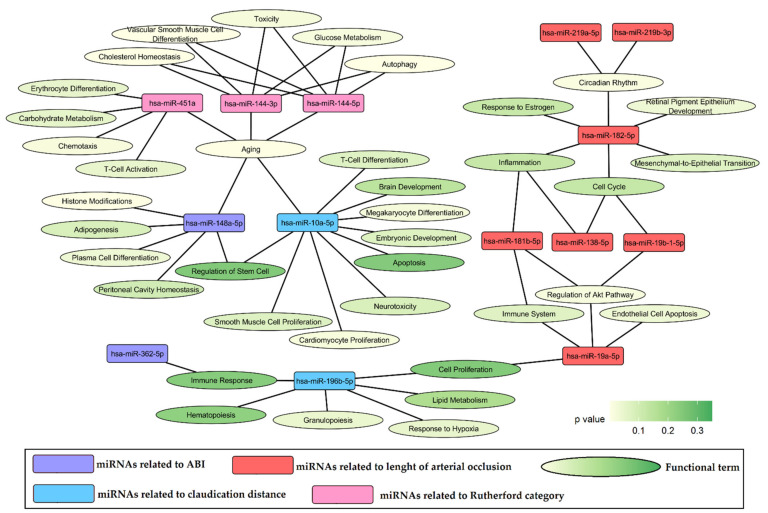
The functional network for selected miRNA sets associated with ABI (ankle brachial index), claudication distance, length of arterial occlusion and Rutherford category in studied group patients with LEAD. The network contains up to 10 the most enriched functional terms disclosed for each miRNA set along with associated miRNAs. Functional relations were obtained from the miRNet 2.0 tool using “miRNA Function” category. *p* value—statistical significance of enrichment.

Functional analysis of miRNAs associated with analyzed indicators of LEAD progression was performed using the miRNet 2.0 online platform. The sets of 2 miRNAs related to ABI, 6 miRNAs related to claudication distance, 11 miRNAs related to the length of arterial occlusion (Figure 1), and 5 miRNAs related to Rutherford category (Table 3, Figure 2) were separately queried to the miRNet 2.0 tool. The functional terms for each set of miRNAs were received using a hypergeometric test applied to the category “miRNA Function”. Up to 10 of the most enriched functional terms (with the lowest *p* value of enrichment) for each set of queried miRNAs as well as associated miRNAs are presented as a network of relationships in Figure 3. Analyzed miRNAs were shown to be associated with various processes underlying vascular pathology, including vascular smooth muscle cell differentiation, endothelial cell apoptosis, response to hypoxia, inflammation, lipid metabolism, circadian rhythm, aging, and others (Figure 3).

### 3.6. Functional Analysis of Genes Regulated by miRNAs Associated with LEAD Indicators

**Figure 4:**
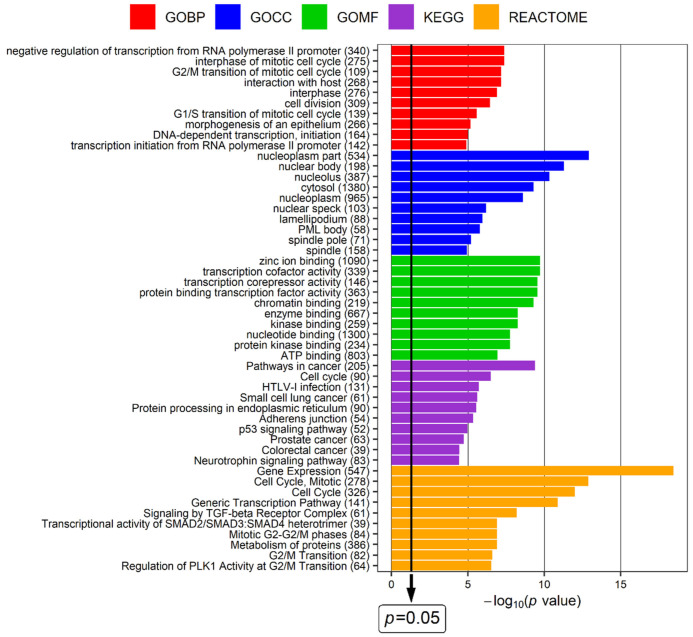
Results of the functional analysis performed using the miRNet 2.0 online platform for genes regulated by 24 miRNAs associated with the studied indicators of LEAD. Up to the top 10 most enriched terms of Gene Ontology Biological Processing (GOBP), Gene Ontology Cellular Compartment (GOCC), Gene Ontology Molecular Function (GOMF), Kyoto Encyclopedia of Genes and Genomes (KEGG), and Reactome categories were presented. *p* value—*p* value for enrichment after adjustment for false discovery rate (FDR), the thick black vertical line represents the *p* = 0.05 threshold. The numbers in brackets following the name of the terms indicate the number of associated genes.

To explore in more detail the biological functions of miRNAs associated with the studied indicators of LEAD, genes targeted by these miRNAs were identified and analyzed in terms of their functions using the miRNet 2.0 online platform. The group of 24 miRNAs, including 2 miRNAs related to ABI, 6 miRNAs related to claudication distance, 11 miRNAs related to the length of arterial occlusion and 5 miRNAs related to Rutherford category, was analyzed with the miRNet tool to identify targeted genes. The analysis revealed 8654 target genes (Supplementary File 2), which were subsequently submitted to functional analysis performed using a hypergeometric test applied to the following categories: KEGG (Kyoto Encyclopedia of Genes and Genomes) pathways, Reactome database, and GO (Gene Ontology) terms including GO Biological Processing, GO Cellular Compartment, and GO Molecular Function subcategories. The top 10 terms with the highest enrichment (with the lowest *p* value of enrichment) resulting from each functional category are presented in Figure 4. The functional terms received from this analysis are mainly related to regulation of cell cycle, regulation of transcription process, and nuclear compartment (Figure 4).

## 4. Discussion

The progression of LEAD results from permanent disturbances in vascular homeostasis, leading to the aggravation of the disease symptoms. Elucidation of molecular mechanisms involved in vascular pathology during various stages of LEAD development could provide new diagnostic, prognostic, and therapeutic options for the disease.

MiRNAs are established factors implicated in LEAD pathology with biomarkers and have therapeutic potential [27,28]. Dysregulations in miRNA expression is a promising research field for determining molecular mechanisms involved in LEAD progression and identifying prognostic markers of LEAD. Therefore, in our study, we implemented a previously generated dataset containing miRNA expression profiles of PBMCs collected from patients with LEAD [34] to identify miRNA expression dysregulations associated with clinical indicators of LEAD (ABI, claudication distance, length of arterial occlusion, Rutherford category, and plaque localization).

Correlation analysis and linear regression were applied to find relationships between miRNA expression and continuous descriptors of LEAD progression, including ABI, claudication distance, and length of arterial occlusion. Two miRNAs (hsa-miR-148a-5p and -362-5p) were found to be relevantly correlated with ABI, six miRNAs (hsa-miR-10a-5p, -196b-5p, -3157-5p, -3182, -32-3p and -941) were found to be relevantly correlated with claudication distance, and eleven miRNAs (hsa-miR-138-5p, -181b-5p, -182-5p, -19a-5p, -19b-1-5p, -219a-5p, -219b-3p, -3620-3p, -429, -548au-5p and -6513-3p) were found to be relevantly correlated with length of occlusion (Table 2, Figure 1). However, selected correlation coefficients associated with these miRNAs were not high and ranged between 0.6 and 0.4, indicating only moderate associations.

Regarding the miRNAs associated with the categorical parameters of LEAD (Rutherford category and plaque localizations), DESeq2 analysis showed 5 miRNAs (hsa-miR-144-3p, -144-5p, -451a, -5100 and -873-5p), which significantly differentiate patients diagnosed with Rutherford category 3 from patients diagnosed with Rutherford category 2 (Table 3, Figure 2). Furthermore, none of the analyzed miRNAs were found to be indicative of the localization of atherosclerotic lesions. Due to significant differences between the numbers of patients in the compared subgroups, presented findings need to be validated in further studies.

The functional analysis of selected miRNAs was performed using the miRNet 2.0 online tool and showed associations with such atherosclerosis-related processes as cellular proliferation, differentiation and death, lipid metabolism, immune response, response to hypoxia, and circadian rhythm (Figure 3). These processes are strongly associated with the pathology of LEAD, indicating the concordance of the obtained results with the studied disease. To further explore the obtained functional associations, functional analysis of genes regulated by selected miRNAs was performed and functional terms closely related to the regulation of the cell cycle, transcription process, and nuclear compartment of cell were determined to be the most enriched (Figure 4).

### 4.1. Comparison with Other miRNAs Previously Reported as Associated with LEAD Progression

Previously, a limited number of studies have focused on the relationships between miRNA expression and LEAD progression. Elevated expression of miR-124-3p in whole blood samples of patients with PAD was found to be negatively correlated with ABI values and was proposed to be associated with PAD severity [38]. Similarly, higher serum levels of miR-93 were found to be positively related to the severity of PAD measured by ABI [41]. Downregulation of miR-126 in plasma was associated with low ABI and the occurrence of symptomatic peripheral artery disease in diabetic patients [42]. The higher expression of miR-21, -92a, -126, and -143 in plasma exosomes was reported to be related to a higher stage of Rutherford category [43]. Furthermore, the expression of miR-130a and -27b in the serum of patients with peripheral atherosclerosis was positively correlated with the Fontaine stages of the disease [30]. None of the abovementioned miRNAs were determined in our study to be associated with the analyzed indicators of LEAD advancement. The presumed reasons for this inconsistency could be significant differences in methodological aspects between studies, including patient inclusion procedures, evaluated characteristics of studied subjects, used biological material, and applied research methods.

In the study by Stather et al., upregulation of miR-196b in whole blood samples was observed in PAD patients compared to control subjects [48]. This finding is consistent with our study, where hsa-miR-196-5p was negatively correlated with the claudication distance, confirming that upregulation of this miRNA is potentially associated with the severity of peripheral atherosclerosis.

### 4.2. Cellular Proliferation and Survival

In our study, higher expression of hsa-miR-138-5p was associated with a smaller size of atherosclerosis plaque (Table 2, Figure 1). Previously, hypoxia-induced elevation of miR-138 expression was observed in endothelial cells and was found to induce endothelial dysfunction, related to reduction in proliferation, angiogenesis, and VEGF-stimulated NO (nitric oxide) production by targeting the *S100A1* gene [49]. This miRNA is downregulated in many types of cancers and primarily acts as a tumor suppressor by targeting many genes associated with cell proliferation, migration, and apoptosis [50,51]. This may suggest that the lower expression of hsa-miR-138-5p associated with larger atherosclerotic lesions (Table 2, Figure 1) indicates a higher proliferative status of atherosclerotic plaques in patients with more advanced LEAD.

The hypothesis that enhanced cell proliferation and survival is responsible for the growth of atherosclerotic lesions could be supported by evidence that many other miRNAs selected in our study are involved in mechanisms regulating these processes. MiR-362-5p, whose higher expression corresponds to lower ABI values and more advanced LEAD (Table 2, Figure 1), exerts an oncogenic effect on acute and chronic myeloid leukemia and is a potential predictor of a worse prognosis of cancer [52,53,54]. Hsa-miR-3157, whose expression is negatively correlated with claudication distance (Table 2, Figure 1), was previously reported to be upregulated in chronic lymphocytic leukemia cells compared to normal B cells [55]. Other miRNAs negatively correlated with claudication distance, hsa-miR-10a-5p and -196b-5p, are involved in vascular endothelial growth factor-stimulated angiogenesis, promoting tumor growth [56]. Finally, decreased expression in endothelial cells of other miRNA positively correlated with length of arterial occlusion, miR-429, previously was associated with the prevention of atherosclerosis by exercise in animal models of atherosclerosis [57]. Other studies reported that this miRNA promotes apoptosis in endothelial cells in vitro and in vivo [58] and inhibits angiogenesis through suppressing endothelial cell proliferation and migration [59].

MiRNAs differentially expressed in PBMCs between groups of patients with LEAD classified according to the Rutherford scale in the 2 and 3 category were also functionally associated with processes related to cell proliferation, differentiation, and death (Figure 3). Two of them, hsa-miR-144-5p and -144-3p, were demonstrated in our study to have higher expression in more advanced LEAD (Table 3, Figure 2) and were previously linked to atherosclerosis. MiR-144-3p exerts pro-atherosclerotic properties by promoting inflammation and regulation of cholesterol homeostasis [60]. The silencing of miR-144 caused a reduction in atherosclerosis in male animal models of this disease [61]. These findings are consistent with studies in human subjects, where elevated circulatory levels of miR-144-3p and miR-144 were observed in patients with acute myocardial infarction [60] and coronary artery disease [62], respectively. Higher expression of miR-144-5p and -144-3p could be associated with LEAD pathology by regulation of proliferation and apoptosis of endothelial cells and VSMCs [63,64].

Other miRNAs related to the Rutherford category, miR-451a, -5100 and -873-5p (Table 3, Figure 2), are also involved in cell proliferation as reported in studies that reveal the role of these miRNAs in cancer [65,66,67,68,69,70,71].

In conclusion, these findings strongly suggest that the imbalance in miRNA-related signals that promote and inhibit cell proliferation and survival is an important factor implicated in the progression of LEAD. This conclusion is further supported by the results of the functional analysis of genes targeted by miRNAs selected in our study, revealing many functional terms closely related to the regulation of the cell cycle (Figure 4).

### 4.3. Response to Hypoxia

Lower expression of hsa-miR-138-5p was found in our study to be negatively correlated with length of atherosclerotic plaque (Table 2, Figure 1). Hu et al. reported downregulation of miR-138 in cardiomyocyte cells injured by hypoxia and reoxygenation events [72]. A similar condition occurs in the lower limbs of patients with LEAD. Repeated ischemia and reperfusion cycles are a primary stimulator of vascular pathology that underly atherosclerosis progression [73,74,75]. Inhibition of this miRNA in cardiomyocytes was linked to aggravated cellular injury by exerting pro-apoptotic effect by targeting Mst1 (mammalian Ste20-like kinase 1) [72]. Therefore, the lower expression of hsa-miR-138-5p found in our study in patients with a longer atherosclerotic plaque (Table 2, Figure 1) could influence disease progression by regulating the apoptosis process triggered by hypoxic conditions.

In our study, hsa-miR-10a-5p was negatively correlated with claudication distance (Table 2, Figure 1), thus its higher expression is potentially related to increased walking impairment in LEAD. MiR-10a belongs to flow-sensitive miRNAs and is upregulated under conditions of normal shear stress, exerting an athero-protective effect by alleviating endothelial dysfunction and inflammation [76]. In the human endothelium of atherosclerotic arteries, the expression level of this miRNA was lower than in normal arteries and was associated with the development of atherosclerotic plaques [77]. These findings are inconsistent with our results, where the higher expression of this miRNA in LEAD patients was correlated with more severe walking impairment (Table 2, Figure 1). This discordance could be explained by a different miR-10a expression pattern in vascular tissues and PBMCs; however, the results of other studies indicate that the higher expression of this miRNA in LEAD could be the result of ischemia and reperfusion injury. As previously observed, miR-10a expression increased significantly after renal ischemia and reperfusion injury in rats and was associated with aggravated renal damage by decreasing cell proliferation and increasing cell apoptosis [78]. A higher exosomal expression of hsa-miR-10a-5p was also found in patients after ischemic events due to intracranial atherosclerotic disease and was associated with a poor response to medical treatments due to inhibition of angiogenesis [79].

### 4.4. Lipids Metabolism and Plaque Stability

Our study demonstrated that miR-148a-5p is negatively correlated with ABI scores (Table 2, Figure 1), thus a higher expression of this miRNA could indicate a greater advance of LEAD. MiR-148a-5p is an important regulator of cholesterol and triglyceride metabolism, and increased expression of this miRNA could contribute to atherosclerosis through increasing LDL levels in blood and controlling cholesterol efflux in macrophages [80]. Previously, elevated serum levels of this miRNA were reported in individuals with metabolic syndrome and were strongly correlated with waist circumference and triglyceride levels in patients with dyslipidemia [81]. Presentations of metabolic syndrome including diabetes, hypertension, dyslipidemia, and obesity are established risk factors for atherosclerosis and LEAD, and upregulation of miR-148a-5p could link metabolic syndrome and atherosclerosis. Furthermore, miR-148a promotes adipocyte differentiation by targeting Wnt1, and an elevated level of this miRNA was proposed as a biomarker of obesity in humans [82]. SNPs in the nucleotide sequence of this miRNA were previously linked to obesity [80]. Taken together, the influence of higher expression of hsa-miR-148a-5p on more progressive LEAD could result in disturbances in lipid metabolism.

Among miRNAs correlated with claudication distance, some are also connected to lipid metabolism. MiR-196b-5p promotes adipogenic differentiation and lipogenesis in progenitor cells through targeting *TSC1* and *TGFBR1* [83]. Increased circulatory levels of hsa-miR-941 were significantly correlated with atherosclerosis risk factors in patients with homozygous familial hypercholesterolemia [84] and were proposed as a biomarker of ST-segment elevation myocardial infarction [85]. Other miRNAs associated with claudication distance are also related to cardiac events: hsa-miR-3182 is a regulator of genes involved in angiogenesis and cardiac muscle cell contraction [86], and increased miR-32-3p circulatory levels have been proposed as a biomarker of severe coronary artery disease [87] and acute ischemic stroke [88]. MiR-32-3p also inhibits ER stress-induced cell apoptosis, increasing atherosclerotic plaque stability [89]. The lower expression of this miRNA related to the shorter claudication distance (Table 2, Figure 1) suggests that a higher instability status of plaques may promote LEAD progression. This supposition could be supported by a negative correlation between the length of atherosclerotic occlusion and the expression of miR-181b-5p (Table 2, Figure 1). Its inhibition was previously reported to increase the stability of atherosclerotic plaques and aneurysm by promoting the production of elastin and collagen [90].

MiR-182 was shown to promote atherosclerosis in animal models by increasing lipid accumulation in atherosclerotic lesions, secretion of proinflammatory cytokines, and activity of lipoprotein lipase [91]. These effects could explain the longer occlusions in LEAD patients who had the higher expression of hsa-miR-182-5p (Table 2, Figure 1). However, other studies have indicated the protective role of hsa-miR-182-5p against atherosclerosis by inhibiting ox-LDL (oxidized low density lipoproteins) stimulated oxidative stress, proliferation of VSMCs, macrophage apoptosis, and foam cell formation [92,93]. Therefore, further studies are needed to elucidate factors influencing the role of hsa-miR-182-5p in atherosclerosis.

### 4.5. Circadian Rhythm

Three miRNAs correlated with the length of arterial occlusion, has-miR-182-5p, -219a-5p, and -219b-3p, were associated with circadian rhythm (Figure 3). Circadian rhythm is involved in many processes implicated in atherosclerosis, including inflammation, lipid metabolism, and functionality of endothelial cells, macrophages, and VSMCs. Much evidence has shown that abnormalities in processes regulating the physiological clock contribute to the development of atherosclerosis [94,95,96]. Circadian rhythm is a complex process regulated by various factors, including miRNAs [97,98]. MiR-219 is one of the circadian-relevant miRNAs [99] and enhanced methylation in this miRNA promoter was found in long-term night shift workers. Circadian rhythm-mediated downregulation of this miRNA probably increases risk of cancer by influence on modulators of cell apoptosis and proliferation [100]. Regarding other circadian rhythm-related miRNAs selected in our study, miR-182 targets *CLOCK*, and dysregulation of this miRNA is a potential reason for circadian rhythm disorder in children with hypoxic ischemic encephalopathy events [101]. To conclude, the altered expression of hsa-miR-182-5p, -219a-5p, and -219b-3p could be implicated in atherosclerosis by modulating circadian-related processes.

The relationships presented between circadian rhythm and atherosclerosis raise the need to characterize patients with atherosclerosis-related disease in terms of sleeping disorders, day or night shift working, traveling between distant time zones, and other features potentially affected circadian rhythm during inclusion in transcriptomic studies.

### 4.6. Vascular Inflammation

Functional networks constructed in our study show that miRNAs correlated with the length of arterial occlusion are mainly linked to terms associated with cell proliferation and inflammation (Figure 3). Among them, hsa-miR-181b-5p was found to be negatively correlated with occlusion size (Table 2, Figure 1), and previous studies demonstrated that miR-181b exerts a protective effect against atherosclerosis by inhibiting endothelial dysfunction and inflammation by targeting Notch1 [102]. This miRNA suppressed shear stress-dependent pyroptosis of endothelial cells by inhibiting the NLRP3 (NLR family pyrin domain containing 3) inflammasome [103]. A higher expression of hsa-miR-181b-5p could influence the size of arterial occlusion by repression of vascular inflammation.

Other miRNAs negatively correlated with the length of arterial occlusion are hsa-miR-19a-5p and hsa-miR-19b-1-5p (Table 2, Figure 1). Both miRNAs target genes with elevated expression in advanced versus primary carotid atherosclerotic plaques [104] and are involved in vascular inflammation during atherosclerosis. In oxidized low-density lipoprotein-stimulated endothelial cells, upregulation of miR-19a is induced by hypoxia-inducible factor (HIF)-1α and is associated with vascular inflammation and atherosclerosis progression [105]. In patients with coronary heart disease, serum miR-19a levels are elevated and contribute to atherosclerosis by promoting vascular inflammation and foam cell formation [106]. The relationship found in this study between the lower expression of this miRNA and larger arterial occlusion may suggest a lower inflammation status of larger atherosclerotic plaques.

Further, miR-19b inhibits leukocyte activation by reducing interleukin 10 expression, and the lowered expression of this miRNA was observed in B cells of atherosclerosis patients [107]. Downregulation of this miRNA was also observed in plasma of patients with coronary artery disease and was related to enhanced tumor necrosis factor (TNF)-α-induced apoptosis in endothelial cells [108]. In contrast to these studies, cell-derived microparticles enriched in miR-19b promote atherosclerotic plaque growth by increasing the burden on lipids, macrophages, and smooth muscle cells with a decrease in collagen content. Interestingly, augmentation of atherosclerosis progression by miR-19b is mediated by inflammation specific for perivascular adipose tissue [109]. These findings suggest tissue-dependent specificity of the pro-inflammatory function of miR-19b in the development of atherosclerosis.

### 4.7. Limitations of the Study

The presented study identified relationships between miRNA expression in PBMCs and progression of LEAD. However, some limitations of this study should be addressed. Due to the descriptive character of our study, all conclusions presented in the Discussion section are hypotheses that need to be validated in further studies. In the studied patient group, there is an imbalance in the number of patients with different Rutherford categories, plaque localization, and other categorical characteristics related to risk factors, medical history, and used medication (Table 1); therefore, the results of the presented work should be considered as having preliminary character and require validation in further studies. Furthermore, a part of selected miRNAs were related not only to the LEAD parameters, but also to other characteristics, including blood parameters (Table 4) as well as status of hypertension, myocardial infarction, and medication with fibrates and metformin (Table 5). Therefore, further validation studies should include a more balanced and much larger group of patients, characterized in detail in terms of risk factors, cardiovascular comorbidities, medical history, blood parameters, and treatment methods.

Our study demonstrated that altered miRNA expression in PBMCs could be useful in evaluating the severity of LEAD. More studies are needed to identify miRNAs with the highest prognostic value to construct predictive models of LEAD progression and to provide targets for the treatment of this disease.

## 5. Conclusions

The presented work demonstrated associations between miRNA expression in PBMCs and parameters of LEAD progression, including ABI, claudication distance, length of arterial occlusion, and Rutherford category, thus showing that miRNA expression could be indicative of LEAD advancement and possess prognostic potential. Functional analysis disclosed that miRNA-related alterations in pathways involved in cell proliferation and survival, response to hypoxia, lipid metabolism, vascular inflammation, and circadian rhythm are potentially responsible for LEAD progression. The presented study shed new light on molecular mechanisms influencing LEAD development, providing potential therapeutic targets for this disease; however, further studies are required to validate our results in larger and more balanced populations.

## Figures and Tables

**Table 1:** Characteristics of 40 LEAD patients included in the study group. The table presents extended data provided in the previous study [34].

Characteristic	Value
**Clinical Parameters of Disease**	
Ankle brachial index (ABI)	0.68 ± 0.05, 0.59–0.8 ^1^
Claudication distance (m)	153.63 ± 33.01, 90–200 ^1^
Length of occlusion (cm)	11.25 ± 5.11, 3–25 ^1^
Rutherford category 2 Rutherford category 3	34 (85%) 6 (15%)
Plaque localization: iliac artery femoral artery iliac and femoral artery popliteal artery femoral and popliteal artery	7 (17.5%) 25 (62.5%) 1 (2.5) 5 (12.5%) 2 (5%)
**Risk Factors and Cardiovascular Comorbidities**	
Age	57.6 ± 9.82, 43–71 ^1^
Sex: male female	35 (87.5%) 5 (12.5%)
Body mass index (BMI)	27.2 ± 2.62, 21.9–31.6 ^1^
Former or current smoker	18 (45%)/22 (55%)
Diabetes type 2	5 (12.5%)
Hypertension	36 (90%)
Coronary artery disease	11 (27.5%)
Myocardial infarction	8 (20%)
Stroke/transient ischemic attack	2 (5%)
**Results of Laboratory Tests**	
Red blood cells (M/µL)	4.74 ± 0.30, 4.11–5.18 ^1^
White blood cells (K/µL)	5.49 ± 0.69, 4.45–6.89 ^1^
Lymphocytes (K/µL)	3.04 ± 0.54, 2.01–3.99 ^1^
Monocytes (K/µL)	0.47 ± 0.15, 0.22–0.87 ^1^
Neutrophils (K/µL)	4.21 ± 0.47, 3.51–5.21 ^1^
Eosinophils (K/µL)	0.21 ± 0.09, 0.10–0.56 ^1^
Basophils (K/µL)	0.10 ± 0.03, 0.07–0.19 ^1^
Platelets (K/µL)	309.3 ± 75.7, 179–561 ^1^
Hemoglobin (g/dL)	14.12 ± 0.52, 12.99–14.99 ^1^
Hematocrit (%)	41.33 ± 1.42, 38.4–43.8 ^1^
Creatinine (mmol/L)	78.70 ± 12.64, 56–99 ^1^
Urea (mmol/L)	4.69 ± 0.83, 2.99–6.02 ^1^
**Medication**	
Statins	34 (85%)
Acetylsalicylic acid	40 (100%)
Clopidogrel	8 (20%)
Beta-adrenergic blockers	27 (67.5%)
Angiotensin-converting enzyme inhibitor	20 (50%)
Ca^2+^ channel blockers	11 (27.5%)
Fibrates	5 (12.5%)
Metformin	2 (5%)
Gliclazide	4 (10%)

^1^ mean ± SD, range.

**Table 2:** Results of the correlation analysis and multivariate linear regression analysis performed between the clinical indicators of LEAD (ABI, claudication distance, and length of occlusion) and miRNA expression in PBMCs of LEAD patients. The table presents results obtained for 19 miRNAs selected from the correlation analysis with the absolute value of Spearman correlation coefficient ≥0.4, *p* value < 0.05, and the average number of normalized counts above 10.

Indicator	miRNA Transcript	miRNA ID ^1^	Correlation Analysis		Regression Analysis	
			R	*p*	Intercept	β
ABI	hsa-mir-148a_hsa-miR-148a-5p	hsa-miR-148a-5p	−0.44	4.26 × 10^−3^	0.831	−0.00174
	hsa-mir-362_hsa-miR-362-5p	hsa-miR-362-5p	−0.40	9.55 × 10^−3^	0.735	−0.000263
Claudication distance	hsa-mir-3182_hsa-miR-3182	hsa-miR-3182	0.49	1.34 × 10^−3^	113.9	0.290
	hsa-mir-10a_hsa-miR-10a-5p	hsa-miR-10a-5p	−0.49	1.47 × 10^−3^	167.0	−0.044
	hsa-mir-32_hsa-miR-32-3p	hsa-miR-32-3p	0.43	6.16 × 10^−3^	75.2	0.568
	hsa-mir-196b_hsa-miR-196b-5p	hsa-miR-196b-5p	−0.42	6.70 × 10^−3^	182.0	−0.581
	hsa-mir-941-4_hsa-miR-941	hsa-miR-941	−0.42	6.84 × 10^−3^	127.1	−1.222
	hsa-mir-3157_hsa-miR-3157-5p	hsa-miR-3157-5p	−0.40	1.05 × 10^−2^	112.9	−2.636
Length of occlusion	hsa-mir-182_hsa-miR-182-5p	hsa-miR-182-5p	0.48	1.82 × 10^−3^	−2.75	0.106
	hsa-mir-19a_hsa-miR-19a-5p	hsa-miR-19a-5p	−0.47	2.01 × 10^−3^	19.11	−0.132
	hsa-mir-3620_hsa-miR-3620-3p	hsa-miR-3620-3p	0.44	4.43 × 10^−3^	3.77	0.259
	hsa-mir-181b-2_hsa-miR-181b-5p	hsa-miR-181b-5p	−0.44	4.51 × 10^−3^	17.57	−0.00128
	hsa-mir-548au_hsa-miR-548au-5p	hsa-miR-548au-5p	−0.43	5.26 × 10^−3^	12.90	−0.499
	hsa-mir-6513_hsa-miR-6513-3p	hsa-miR-6513-3p	0.42	6.28 × 10^−3^	9.47	0.264
	hsa-mir-19b-1_hsa-miR-19b-1-5p	hsa-miR-19b-1-5p	−0.42	6.39 × 10^−3^	18.26	−0.045
	hsa-mir-138-1_hsa-miR-138-5p	hsa-miR-138-5p	−0.42	7.50 × 10^−3^	20.57	−0.184
	hsa-mir-429_hsa-miR-429	hsa-miR-429	0.41	8.88 × 10^−3^	11.98	0.135
	hsa-mir-219a-2_hsa-miR-219a-5p	hsa-miR-219a-5p	−0.40	1.03 × 10^−2^	20.19	−0.122
	hsa-mir-219b_hsa-miR-219b-3p	hsa-miR-219b-3p	−0.40	1.03 × 10^−2^	20.19	−0.122

^1^ according to miRBase 22.1 (http://www.mirbase.org), *p*—statistical significance of correlation, R—Spearman correlation coefficient, β—regression coefficient adjusted by age, sex, BMI and smoking. MiRNA transcripts were ordered inside each group according to decreasing absolute values of Spearman correlation coefficients.

**Table 3:** MiRNA transcripts found to be differentially expressed in PBMCs of LEAD patients diagnosed with Rutherford category 3 compared to LEAD patients with Rutherford category 2. The table presents the miRNAs resulted from the DESeq2 analysis with Benjamini-Hochberg FDR < 0.05.

miRNA Transcript	miRNA ID ^1^	*p*	Fold Change	ROC-AUC
hsa-mir-144_hsa-miR-144-3p	hsa-miR-144-3p	1.256 × 10^−10^	12.435	0.843
hsa-mir-451a_hsa-miR-451a	hsa-miR-451a	5.855 × 10^−10^	9.546	0.892
hsa-mir-144_hsa-miR-144-5p	hsa-miR-144-5p	2.784 × 10^−6^	5.016	0.824
hsa-mir-5100_hsa-miR-5100	hsa-miR-5100	1.076 × 10^−2^	2.062	0.711
hsa-mir-873_hsa-miR-873-5p	hsa-miR-873-5p	1.388 × 10^−2^	0.261	0.892

^1^ according to miRBase 22.1 (http://www.mirbase.org). The table presents *p* (FDR with Benjamini-Hochberg correction) and fold change values received from the DESeq2 analysis as well as areas under ROC curves (ROC-AUC) received from the ROC analysis. MiRNA transcripts were ordered according to increasing *p* value.

**Table 4:** Results of the correlation analysis performed between 24 miRNAs selected as potentially associated with LEAD indicators and continuous characteristics of the study group. The table presents the correlations obtained with the absolute value of the Spearman correlation coefficient ≥0.4 and *p* value < 0.05.

Characteristic	miRNA Transcript	miRNA ID ^1^	R	*p*
Basophils	hsa-mir-138-1_hsa-miR-138-5p	hsa-miR-138-5p	0.45	3.935 × 10^−3^
	hsa-mir-19b-1_hsa-miR-19b-1-5p	hsa-miR-19b-1-5p	0.40	9.865 × 10^−3^
	hsa-mir-181b-2_hsa-miR-181b-5p	hsa-miR-181b-5p	0.40	1.002 × 10^−2^
Eosinophils	hsa-mir-144_hsa-miR-144-5p	hsa-miR-144-5p	−0.45	3.850 × 10^−3^
	hsa-mir-3157_hsa-miR-3157-5p	hsa-miR-3157-5p	0.41	9.200 × 10^−3^
	hsa-mir-3620_hsa-miR-3620-3p	hsa-miR-3620-3p	−0.40	9.839 × 10^−3^
Monocytes	hsa-mir-19b-1_hsa-miR-19b-1-5p	hsa-miR-19b-1-5p	0.66	3.432 × 10^−6^
	hsa-mir-181b-2_hsa-miR-181b-5p	hsa-miR-181b-5p	0.43	5.291 × 10^−3^
	hsa-mir-548au_hsa-miR-548au-5p	hsa-miR-548au-5p	0.43	5.358 × 10^−3^
Red blood cells	hsa-mir-5100_hsa-miR-5100	hsa-miR-5100	−0.41	9.129 × 10^−3^
Hemoglobin	hsa-mir-5100_hsa-miR-5100	hsa-miR-5100	−0.42	7.436 × 10^−3^

^1^ according to miRBase 22.1 (http://www.mirbase.org), R—Spearman correlation coefficient, *p*—statistical significance.

**Table 5:** Results of the differential expression analysis of 24 miRNAs selected as potentially associated with LEAD indicators, performed between subgroups of patients with different status of analyzed categorical characteristics. The table presents the miRNAs resulted from the DESeq2 analysis with Benjamini-Hochberg FDR < 0.05.

Characteristic	miRNA Transcript	miRNA ID ^1^	*p*	Fold Change
Hypertension	hsa-mir-5100_hsa-miR-5100	hsa-miR-5100	4.823 × 10^−4^	0.444
Myocardial infarction	hsa-mir-219a-2_hsa-miR-219a-5p	hsa-miR-219a-5p	4.396 × 10^−2^	0.493
	hsa-mir-219b_hsa-miR-219b-3p	hsa-miR-219b-3p	4.396 × 10^−2^	0.493
Medication with fibrates	hsa-mir-144_hsa-miR-144-3p	hsa-miR-144-3p	4.872 × 10^−9^	11.014
	hsa-mir-451a_hsa-miR-451a	hsa-miR-451a	7.336 × 10^−7^	7.602
	hsa-mir-144_hsa-miR-144-5p	hsa-miR-144-5p	6.029 × 10^−6^	4.415
	hsa-mir-362_hsa-miR-362-5p	hsa-miR-362-5p	1.280 × 10^−2^	0.327
Medication with metformin	hsa-mir-362_hsa-miR-362-5p	hsa-miR-362-5p	5.750 × 10^−3^	0.122

^1^ according to miRBase 22.1 (http://www.mirbase.org). The table presents *p* (FDR with Benjamini-Hochberg correction) and fold change values received from DESeq2 analysis.

## Data Availability

The expression data used for this study are openly available in FigShare repository at https://doi.org/10.6084/m9.figshare.19164659.v2, accessed on 11 February 2022.

## Supplementary Materials

The following supporting information can be downloaded at: https://www.mdpi.com/article/10.3390/jcm11061619/s1, Figure S1: Scatter plots of ankle brachial index (ABI) and expression of 9 miRNAs correlated with this parameter with the absolute value of Spearman correlation coefficient ≥0.4, Figure S2: Scatter plots of claudication distances and expression of 18 miRNAs correlated with this parameter with the absolute value of Spearman correlation coefficient ≥0.4, Figure S3: Scatter plots of length of arterial occlusion and expression of 21 miRNAs correlated with this parameter with the absolute value of Spearman correlation coefficient ≥0.4, Figure S4: Quality control plots generated for the results of the differential miRNA expression analysis performed by the DESeq2 package between 6 LEAD patients diagnosed with Rutherford category 3 and 34 patients diagnosed with Rutherford category 2, Figure S5: Receiver operating characteristics (ROC) plots performed for 5 differentially expressed miRNAs in 6 LEAD patients diagnosed with Rutherford category 3 versus 34 patients diagnosed with Rutherford category 2, Figure S6: Scatter plots depicting selected correlations between miRNAs and the continuous characteristics, Figure S7: Boxplots presenting the expression of selected miRNAs in patients with different status of the categorical characteristics, Table S1: Results of the correlation analysis and multivariate linear regression analysis performed between the clinical characteristics of LEAD (ABI, claudication distance and length of occlusion) and miRNA expression in PBMCs of LEAD patients, Table S2: Detailed results of the ROC analysis for five differentially expressed miRNAs in 6 LEAD patients diagnosed with Rutherford category 3 versus 34 patients diagnosed with Rutherford category 2, Table S3: Results of correlation analysis performed between ankle/brachial index (ABI) and miRNA expression in PBMCs of patients with lower extremity artery disease, Table S4: Results of correlation analysis performed between claudication distance and miRNA expression in PBMCs of patients with lower extremity artery disease, Table S5: Results of correlation analysis performed between length of arterial occlusion and miRNA expression in PBMCs of patients with lower extremity artery disease, Table S6: Results of differential miRNA expression analysis performed using DESeq2 method between 6 lower extremity artery disease (LEAD) patients diagnosed with Rutherford category 3 versus 34 LEAD patients diagnosed with Rutherford category 2, Table S7: Results of differential miRNA expression analysis performed using DESeq2 method between 7 lower extremity artery disease (LEAD) patients with iliac plaques and 25 LEAD patients with femoral plaques, Table S8: Results of differential miRNA expression analysis performed using DESeq2 method between 7 lower extremity artery disease (LEAD) patients with iliac plaques and 5 LEAD patients with popliteal plaques, Table S9: Results of differential miRNA expression analysis performed using DESeq2 method between 5 lower extremity artery disease (LEAD) patients with popliteal plaques and 25 LEAD patients with femoral plaques, Table S10: Targets for miRNAs related to lower extremity artery disease, identified using miRNet 2.0 tool.
