# Supplementary material for: Relationships between Indicators of Lower Extremity Artery Disease and miRNA Expression in Peripheral Blood Mononuclear Cells
Source: J Clin Med. 2022 Mar 15;11(6):1619. doi: 10.3390/jcm11061619 (PMC8948757; doi:10.3390/jcm11061619)
Supplement: Supplementary file 1 [file jcm-11-01619-s001.zip › miRNA_prognose_LEAD_Supplementary_File_1.pdf]

SUPPLEMENTARY MATERIAL

Supplementary Figures

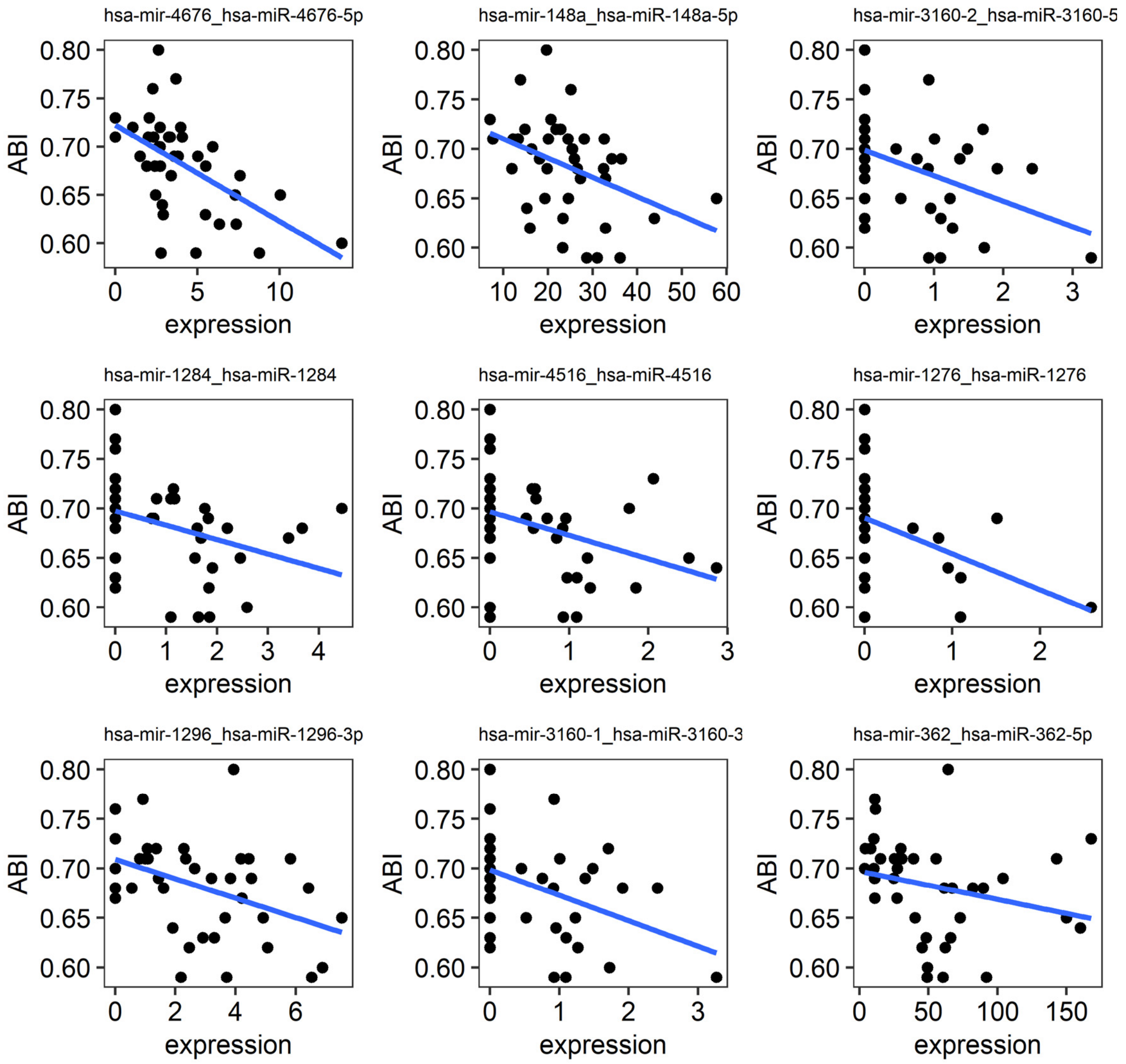

Figure S1. Scatter plots of ankle brachial index (ABI) and expression of 9 miRNAs correlated with this parameter with the absolute value of Spearman correlation coefficient ≥ 0.4. The blue lines indicate the fitting line of linear regression model.

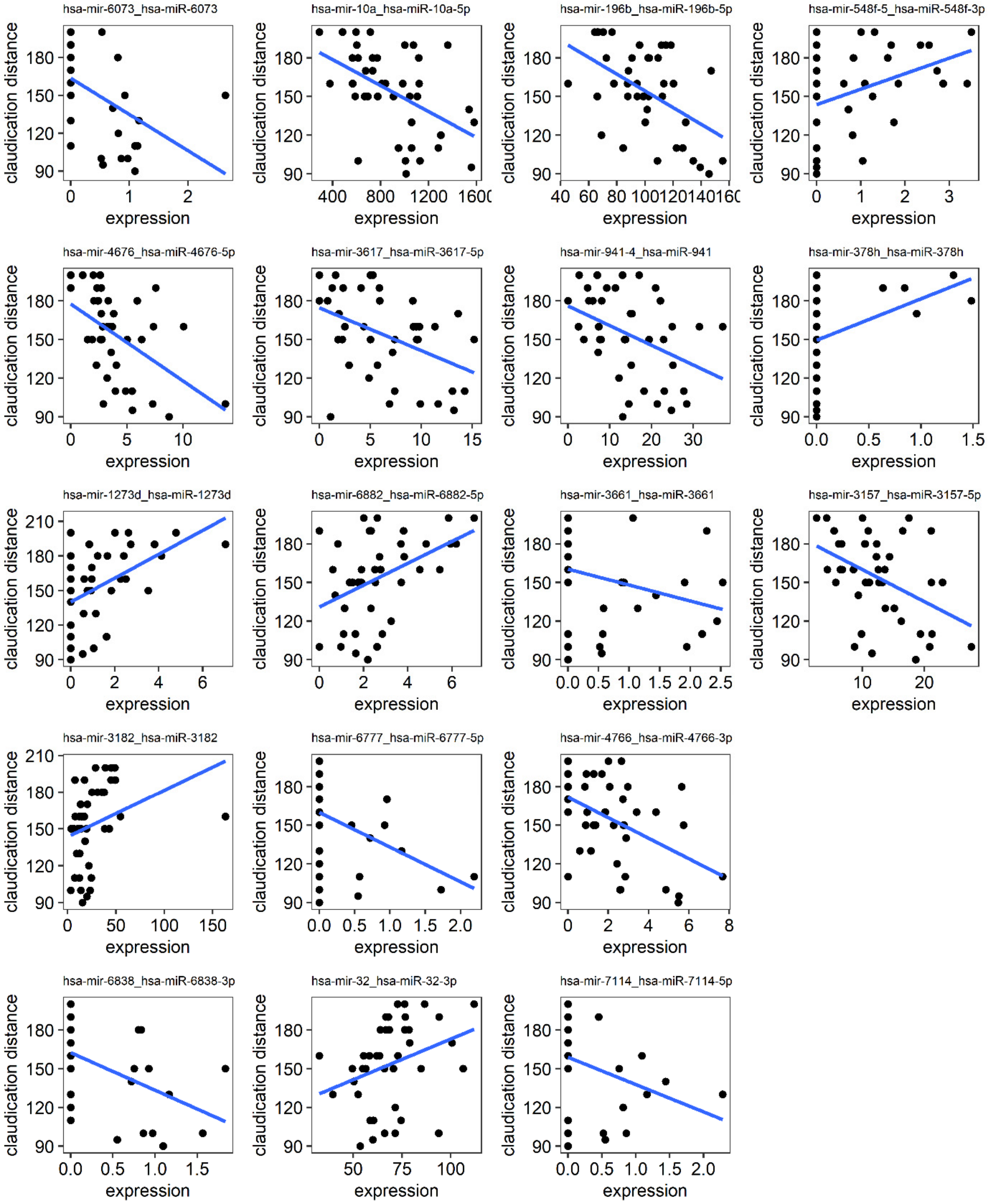

Figure S2. Scatter plots of claudication distances and expression of 18 miRNAs correlated with this parameter with the absolute value of Spearman correlation coefficient ≥ 0.4. The blue lines indicate the fitting line of linear regression model.

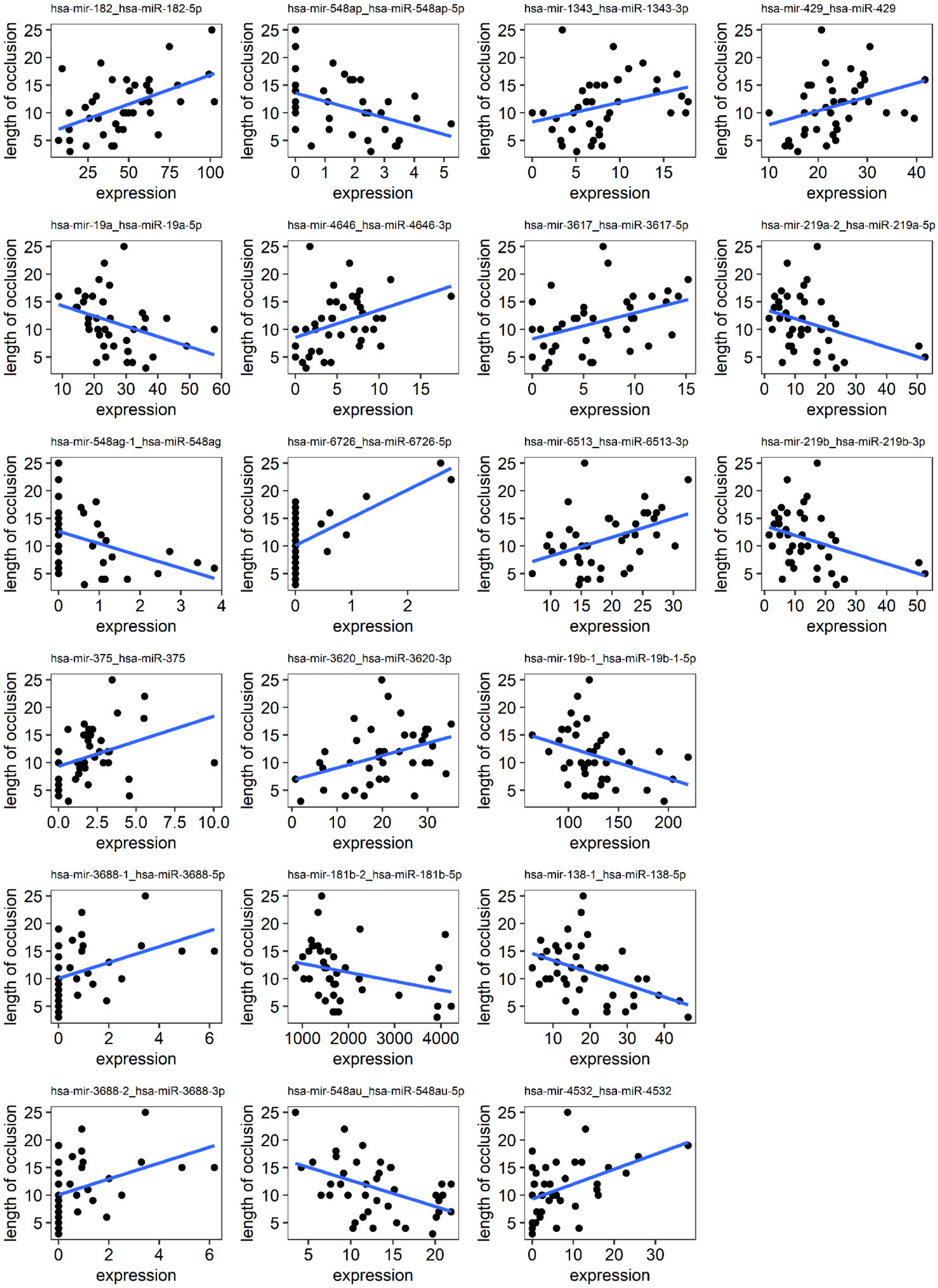

Figure S3. Scatter plots of length of arterial occlusion and expression of 21 miRNAs correlated with this parameter with the absolute value of Spearman correlation coefficient ≥ 0.4. The blue lines indicate the fitting line of linear regression model.

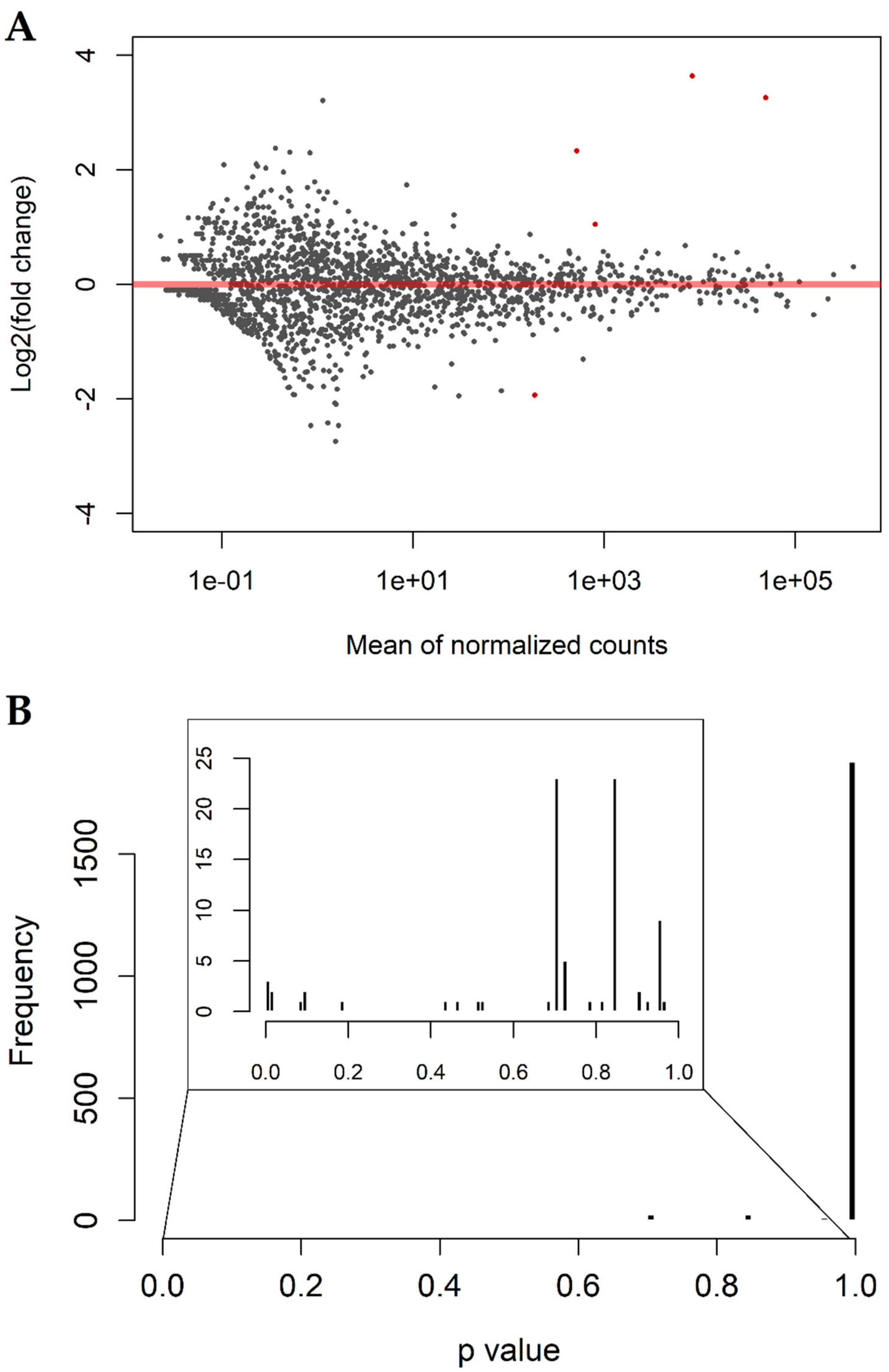

Figure S4. Quality control plots generated for the results of the differential miRNA expression analysis performed by the DESeq2 package between 6 LEAD patients diagnosed with Rutherford category 3 and 34 patients diagnosed with Rutherford category 2. (**A**) MA plot showing log2 fold changes of differentially expressed miRNAs against means of normalized counts. The points of miRNAs with Benjamini-Hochberg FDR < 0.05 were marked in red. (**B**) Distribution of Benjamini-Hochberg FDR values. The rectangle shows magnification of the frequencies regarding FDR values in range from 0 to 0.99.

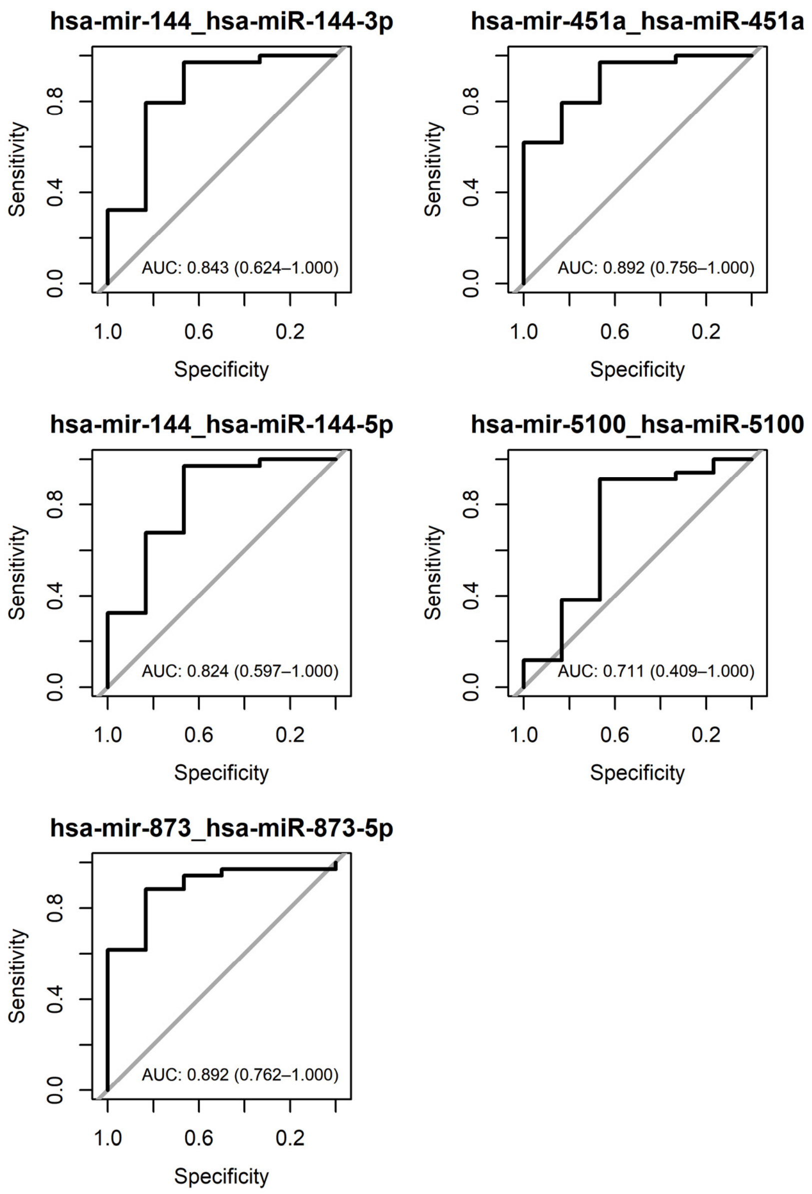

Figure S5. Receiver Operating Characteristics (ROC) plots performed for 5 differentially expressed miRNAs in 6 LEAD patients diagnosed with Rutherford category 3 versus 34 patients diagnosed with Rutherford category 2. The values of the area under curves (AUC) with a 95% confidence interval (in brackets) were included in each plot.

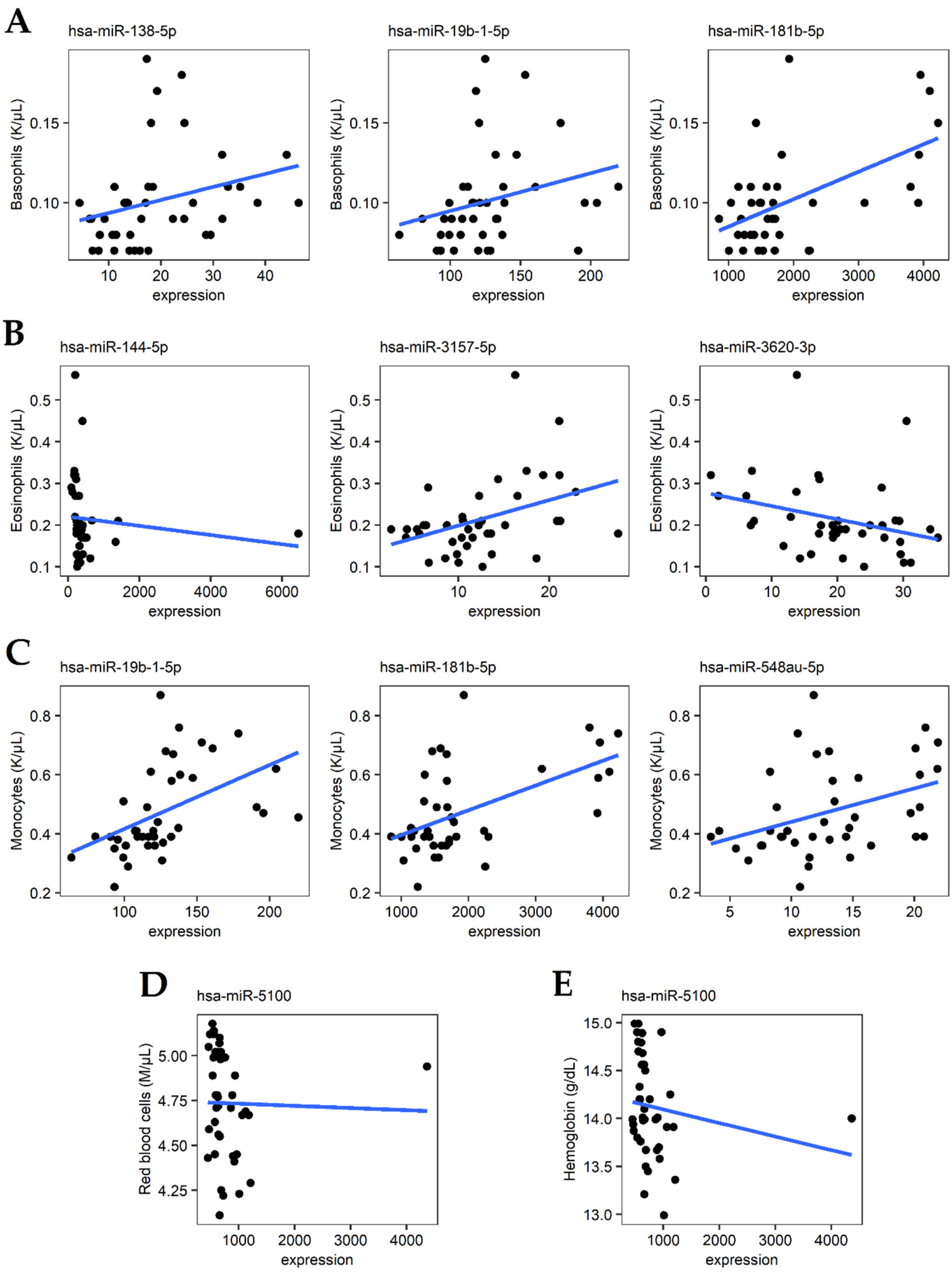

Figure S6. Scatter plots depicting selected correlations between miRNAs and the continuous characteristics: **A)** basophil counts, **B)** eosinophil counts, **C)** monocyte counts, **D)** red blood cell counts, and **E)** hemoglobin levels. The blue lines indicate the trend line (the fitting line of the simple linear regression model).

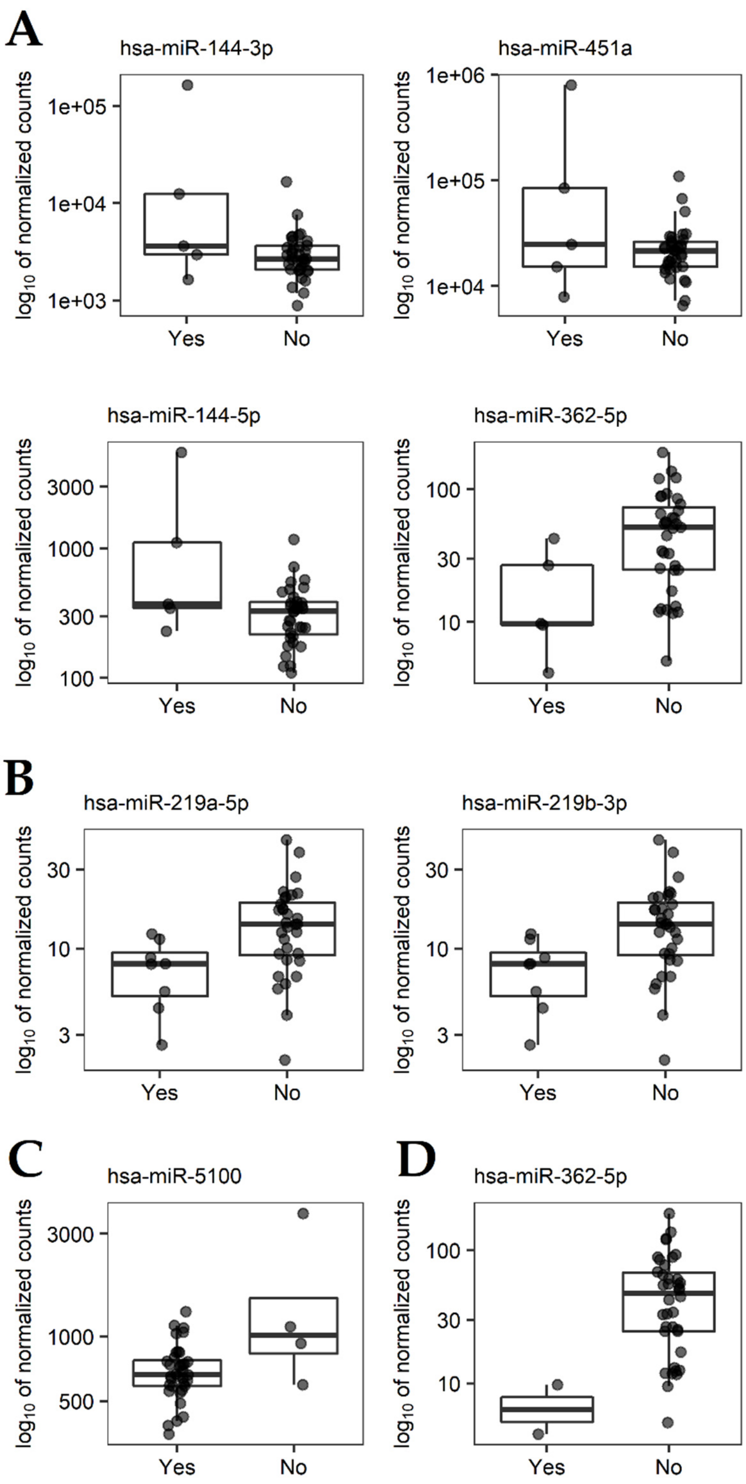

Figure S7. Boxplots presenting the expression of selected miRNAs in patients with different status of the categorical characteristics: **A)** medication with fibrates, **B)** myocardial infarction, **C)** hypertension, and **D)** medication with metformin. In the boxplots, whiskers reach extreme samples inside the 1.5 interquartile range, boxes range between 25% and 75% quartile, and horizontal lines inside boxes mark median values.

Supplementary Tables

Table S1. Results of the correlation analysis and multivariate linear regression analysis performed between the clinical characteristics of LEAD (ABI, claudication distance and length of occlusion) and miRNA expression in PBMCs of LEAD patients. The table presents miRNAs selected with the absolute value of Spearman correlation coefficient ≥ 0.4 and *p* value < 0.05 (the entire correlation results are provided in Supplementary File 2).

Characteristic	miRNA transcript	miRNA ID [1]	Correlation analysis		Regression line		Selected as relevant? [4]
			R	*p*	Intercept	β	
ABI	hsa-mir-4676_hsa-miR-4676-5p	hsa-miR-4676-5p	-0.55	2.83×10^{-2}	0.802	-1.03×10^{-2}	no
	hsa-mir-1284_hsa-miR-1284	hsa-miR-1284	-0.48	1.76×10^{-3}	0.723	-1.60×10^{-2}	no
	hsa-mir-1296_hsa-miR-1296-3p	hsa-miR-1296-3p	-0.44	4.12×10^{-3}	0.750	-8.33×10^{-3}	no
	hsa-mir-148a_hsa-miR-148a-5p	hsa-miR-148a-5p	-0.44	4.26×10^{-3}	0.831	-1.74×10^{-3}	yes
	hsa-mir-4516_hsa-miR-4516	hsa-miR-4516	-0.43	5.42×10^{-3}	0.735	-2.26×10^{-2}	no
	hsa-mir-3160-1_hsa-miR-3160-3p	hsa-miR-3160-3p	-0.43	5.95×10^{-3}	0.777	-2.13×10^{-2}	no
	hsa-mir-3160-2_hsa-miR-3160-5p	hsa-miR-3160-5p	-0.43	5.95×10^{-3}	0.777	-2.13×10^{-2}	no
	hsa-mir-1276_hsa-miR-1276	hsa-miR-1276	-0.41	8.90×10^{-3}	0.806	-3.94×10^{-2}	no
	hsa-mir-362_hsa-miR-362-5p	hsa-miR-362-5p	-0.40	9.55×10^{-3}	0.735	-2.63×10^{-4}	yes
Claudication distance	hsa-mir-6073_hsa-miR-6073	hsa-miR-6073	-0.59	1.02×10^{-2}	136.5	-25.673	no
	hsa-mir-4676_hsa-miR-4676-5p	hsa-miR-4676-5p	-0.52	4.88×10^{-2}	132.9	-6.551	no
	hsa-mir-1273d_hsa-miR-1273d	hsa-miR-1273d [2]	0.52	6.07×10^{-4}	71.8	9.470	no
	hsa-mir-3182_hsa-miR-3182	hsa-miR-3182	0.49	1.34×10^{-3}	113.9	0.290	yes
	hsa-mir-6838_hsa-miR-6838-3p	hsa-miR-6838-3p	-0.49	1.41×10^{-3}	91.1	-28.896	no
	hsa-mir-10a_hsa-miR-10a-5p	hsa-miR-10a-5p	-0.49	1.47×10^{-3}	167.0	-0.044	yes
	hsa-mir-3617_hsa-miR-3617-5p	hsa-miR-3617-5p	-0.46	2.97×10^{-3}	155.8	-2.681	no
	hsa-mir-6882_hsa-miR-6882-5p	hsa-miR-6882-5p	0.44	4.84×10^{-3}	127.5	8.125	no
	hsa-mir-6777_hsa-miR-6777-5p	hsa-miR-6777-5p	-0.43	5.20×10^{-3}	125.3	-21.022	no
	hsa-mir-32_hsa-miR-32-3p	hsa-miR-32-3p	0.43	6.16×10^{-3}	75.2	0.568	yes
	hsa-mir-196b_hsa-miR-196b-5p	hsa-miR-196b-5p	-0.42	6.70×10^{-3}	182.0	-0.581	yes
	hsa-mir-941-4_hsa-miR-941	hsa-miR-941	-0.42	6.84×10^{-3}	127.1	-1.222	yes
	hsa-mir-3661_hsa-miR-3661	hsa-miR-3661	-0.42	6.92×10^{-3}	148.9	-11.511	no
	hsa-mir-4766_hsa-miR-4766-3p	hsa-miR-4766-3p	-0.41	8.22×10^{-3}	136.4	-6.707	no
	hsa-mir-7114_hsa-miR-7114-5p	hsa-miR-7114-5p	-0.41	8.57×10^{-3}	154.6	12.513	no
	hsa-mir-548f-5_hsa-miR-548f-3p	hsa-miR-548f-3p	0.41	9.33×10^{-3}	151.6	-24.836	no
	hsa-mir-378h_hsa-miR-378h	hsa-miR-378h	0.40	1.00×10^{-2}	119.6	25.319	no
	hsa-mir-3157_hsa-miR-3157-5p	hsa-miR-3157-5p	-0.40	1.05×10^{-2}	112.9	-2.636	yes
Length of occlusion	hsa-mir-182_hsa-miR-182-5p	hsa-miR-182-5p	0.48	1.82×10^{-3}	-2.75	0.106	yes
	hsa-mir-19a_hsa-miR-19a-5p	hsa-miR-19a-5p	-0.47	2.01×10^{-3}	19.11	-0.132	yes
	hsa-mir-548ag-1_hsa-miR-548ag	hsa-miR-548ag	-0.46	2.64×10^{-3}	18.92	-2.156	no
	hsa-mir-375_hsa-miR-375	hsa-miR-375	0.45	3.44×10^{-3}	22.23	0.790	no
	hsa-mir-3688-1_hsa-miR-3688-5p	hsa-miR-3688-5p	0.45	3.55×10^{-3}	11.86	1.422	no
	hsa-mir-3688-2_hsa-miR-3688-3p	hsa-miR-3688-3p	0.45	3.55×10^{-3}	11.86	1.422	no
	hsa-mir-548ap_hsa-miR-548ap-5p	hsa-miR-548ap-5p	-0.45	3.58×10^{-3}	23.01	-1.431	no
	hsa-mir-4646_hsa-miR-4646-3p	hsa-miR-4646-3p	0.45	3.96×10^{-3}	12.18	0.408	no
	hsa-mir-6726_hsa-miR-6726-5p	hsa-miR-6726-5p	0.44	4.20×10^{-3}	17.98	4.420	no
	hsa-mir-3620_hsa-miR-3620-3p	hsa-miR-3620-3p	0.44	4.43×10^{-3}	3.77	0.259	yes
	hsa-mir-181b-2_hsa-miR-181b-5p	hsa-miR-181b-5p	-0.44	4.51×10^{-3}	17.57	-1.275×10^{-3}	yes
	hsa-mir-548au_hsa-miR-548au-5p	hsa-miR-548au-5p	-0.43	5.26×10^{-3}	12.90	-0.499	yes
	hsa-mir-1343_hsa-miR-1343-3p	hsa-miR-1343-3p	0.43	5.59×10^{-3}	17.23	0.399	no
	hsa-mir-3617_hsa-miR-3617-5p	hsa-miR-3617-5p	0.43	6.10×10^{-3}	13.83	0.350	no
	hsa-mir-6513_hsa-miR-6513-3p	hsa-miR-6513-3p	0.42	6.28×10^{-3}	9.47	0.264	yes
	hsa-mir-19b-1_hsa-miR-19b-1-5p	hsa-miR-19b-1-5p	-0.42	6.39×10^{-3}	18.26	-0.045	yes
	hsa-mir-138-1_hsa-miR-138-5p	hsa-miR-138-5p	-0.42	7.50×10^{-3}	20.57	-0.184	yes
	hsa-mir-4532_hsa-miR-4532	hsa-miR-4532 [3]	0.41	8.05×10^{-3}	20.77	0.305	no
	hsa-mir-429_hsa-miR-429	hsa-miR-429	0.41	8.88×10^{-3}	11.98	0.135	yes
	hsa-mir-219a-2_hsa-miR-219a-5p	hsa-miR-219a-5p	-0.40	1.03×10^{-2}	20.19	-0.122	yes
	hsa-mir-219b_hsa-miR-219b-3p	hsa-miR-219b-3p	-0.40	1.03×10^{-2}	20.19	-0.122	yes

[1] according to miRBase 22.1 (http://www.mirbase.org), [2] hsa-miR-1273d was reported by miRBase 22.1 as a "dead" miRNA being considered as derived from signal recognition particle (SRP) RNA or derived from *Alu* RNA, [3] hsa-miR-4532 was reported by miRBase 22.1 as a "dead" miRNA being considered as a fragment of 28S rRNA, [4] miRNAs with the average number of normalized counts >10 were considered as relevant, R - Spearman correlation coefficient, p - statistical significance of correlation, β – regression coefficient adjusted by age, gender, BMI and smoking. MiRNA transcripts were ordered inside each group according to decreasing absolute values of Spearman correlation coefficients.

Table S2. Detailed results of the ROC analysis for five differentially expressed miRNAs in 6 LEAD patients diagnosed with Rutherford category 3 versus 34 patients diagnosed with Rutherford category 2.

miRNA transcript	ROC-AUC[1]	Threshold	Specificity	Sensitivity	Accuracy	Positive Predictive Value	Negative Predictive Value
hsa-mir-451a_hsa-miR-451a	0.892	40497.128	0.667	0.971	0.925	0.943	0.800
hsa-mir-873_hsa-miR-873-5p	0.892	66.968	0.833	0.882	0.875	0.968	0.556
hsa-mir-144_hsa-miR-144-3p	0.843	4857.331	0.667	0.971	0.925	0.943	0.800
hsa-mir-144_hsa-miR-144-5p	0.824	568.602	0.667	0.971	0.925	0.943	0.800
hsa-mir-5100_hsa-miR-5100	0.711	950.645	0.667	0.912	0.875	0.939	0.571

[1]Area under ROC curve.
